# Microarray profiling identifies extracellular circulating miRNAs dysregulated in cystic fibrosis

**DOI:** 10.1038/s41598-019-51890-7

**Published:** 2019-10-29

**Authors:** Justin E. Ideozu, Xi Zhang, Vittobai Rangaraj, Susanna McColley, Hara Levy

**Affiliations:** 1 Ann & Robert H. Lurie Children’s Hospital of Chicago, Chicago, IL 60611 USA; 2 Human Molecular Genetics Program, Stanley Manne Children’s Research Institute, Chicago, IL 60614 USA; 3 Feinberg School of Medicine at Northwestern University Chicago, Chicago, IL 60611 USA

**Keywords:** miRNAs, Transcriptomics

## Abstract

Extracellular circulating miRNAs (ECmiRNAs) play a crucial role in cell-to-cell communication and serve as non-invasive biomarkers in a wide range of diseases, but their abundance and functional relevance in cystic fibrosis (CF) remain poorly understood. In this study, we employed microarray technology to identify aberrantly expressed plasma ECmiRNAs in CF and elucidate the functional relevance of their targets. Overall, we captured several ECmiRNAs abundantly expressed in CF. Expression levels of 11 ECmiRNAs differed significantly between CF and healthy control (HC) samples (FDR < 0.05, log2 FC≥2). Among these, 10 were overexpressed while only hsa-miR-598-3p was underexpressed in CF. The overexpressed miRNAs included three let-7 family members (hsa-let-7b-5p, hsa-let-7c-5p and hsa-let-7d-5p), three 103/107 family members (hsa-mir-103a-3p; hsa-mir-103b; hsa-mir-107), hsa-miR-486-5p, and other miRNAs. Using in silico methods, we identified 2,505 validated targets of the 11 differentially expressed miRNAs. Hsa-let-7b-5p was the most important hub in the network analysis. The top-ranked validated targets were involved in miRNA biogenesis and gene expression, including *AGO1*, *DICER1*, *HMGA1*, and *MYC*. The top pathways influenced by all targets were primarily signal transduction pathways associated with CF, including PI3K/Akt-, Wnt/β catenin-, glucocorticoid receptor-, and mTor signaling pathways. Our results suggest ECmiRNAs may be clinically relevant in CF and warrant further study.

## Introduction

Cystic fibrosis (CF) is a multisystem genetic disease caused by mutations in the cystic fibrosis conductance regulator (*CFTR*) gene^1,2^. Recent advances in molecular methods have led to the identification of over 2000 CF-causing variants (http://www.genet.sickkids.on.ca/), but phenotypic variability presented among patients with the same *CFTR* genotype remains a major therapeutic challenge^3,4^. This has driven an intense search for novel molecular drivers relevant to CF pathophysiology that may hold promise as biomarkers or therapeutic targets^5,6^.

Accumulating evidence suggests a plethora of cellular microRNAs (miRNAs) are dysregulated in CF^7–10^ and may characterize its lung phenotypes^11,12^. miRNAs are small non-coding RNA species (~20–25 nt in length) that regulate 30% of human genes and numerous fundamental biological processes^13,14^. Because their expression levels can be modulated *in vivo* and *in vitro* to mediate the expression of their target genes^15^, including *CFTR*^16^, they are promising therapeutic targets for human diseases. Interestingly, miRNA dysregulation has been associated with several other human diseases including various cancers^17,18^ and chronic respiratory diseases^19^. Although much insight about miRNA dysregulation in CF has been gained from studying epithelial^7–9^ and CF plasma-induced blood cells^12^, knowledge of extracellular miRNA abundance and expression remains poorly understood.

Extracellular circulating miRNAs (ECmiRNAs) are present outside their parental cells and have been detected in many biological fluids including blood plasma and serum^20^. In order to exist stably in the extracellular environment and exert their function, miRNAs secreted from parental cells are encapsulated within extracellular vesicles, including exosomes, or bound to proteins and lipids^20,21^. ECmiRNAs play a crucial role in promoting intercellular signaling and are capable of influencing the expression of their target genes in recipient cells^20,22^. ECmiRNA expression profiles may correlate with changes in cellular signaling events and can be disease-specific^23^. Altered expression levels of ECmiRNAs have been reported in many human diseases^24^. In CF, the diagnostic potential of serum miRNAs for liver disease have been demonstrated^25^, but there is sparse literature on CF-relevant plasma ECmiRNAs.

In this study, we utilized microarray-based technology to identify plasma ECmiRNAs differentially expressed between CF and healthy controls (HC), and to characterize the functional relevance of their mRNA targets. We report that several miRNAs are abundantly expressed in CF plasma. Among these, 11 miRNAs were significantly differentially expressed between CF and HC samples. In silico analysis revealed that the top-ranked validated targets of the differentially expressed miRNAs were genes involved in miRNA biogenesis and gene expression, and the top-ranked pathways influenced by all the validated targets were primarily within signal transduction pathways known to be involved in CF pathogenesis.

## Results

### Baseline characteristics of study samples

We performed miRNA microarray profiling to identify ECmiRNAs differentially expressed between CF and HC plasma samples. Clinical and demographic information for the CF patients and HCs who provided samples are shown in Table 1. The mean age (±SD) for CF patients whose samples were used in the discovery and validation phases were 16.6 ± 4.8 and 22.3 ± 4.4, respectively. CF samples were evenly split between male and female patients. The mean (±SD) sweat chloride level was 101. ± 3.1 for CF patients in the discovery group and 103.2 ± 3.6 for those in the validation group. Analysis of pulmonary function test data showed the CF patients in the discovery group had a mean (±SD) FEV_1_% predicted of 70.4 ± 18.5, while 84 ± 22.6 was recorded for those in the validation cohort. The HC samples had a mean age (±SD) of 22.8 ± 2.6, with males accounting for 70% of the samples. Overall, no significant differences in demographic and clinical data were observed between samples in discovery and validation group (Table 1).

**Table 1:** Demographic and clinical characteristics of the study cohort.

Status	Clinical parameters	Discovery cohort	Validation cohort	*P* value^a^
Cysticfibrosis	**Number (n)**	**5**	**5**	Not significant
	Age in years, mean ± SD	16.6 ± 4.8	22.3 ± 4.4	Not significant
	Gender (Male:Female)	2:3	3:2	Not significant
	Sweat chloride, mean ± SD	101.6 ± 3.1	103.2 ± 3.6	Not significant
	FEV_1_ % predicted, mean ± SD	70.4 ± 18.5	84 ± 22.6	Not significant
Healthy controls	**Number (n)**	**5**	**5**	Not significant
	Age in years, mean (SD)	23 ± 1.6	22.6 ± 3.5	Not significant
	Gender (Male:Female)	5:0	2:3	Not significant

^a^*P* value was estimated using a *t-test*. All CF samples were homozygous for DF508del *CFTR* mutation and negative for mucoid *Pseudomonas aeruginosa infection*.

### Quality assessment of microarray data

**Figure 1:**
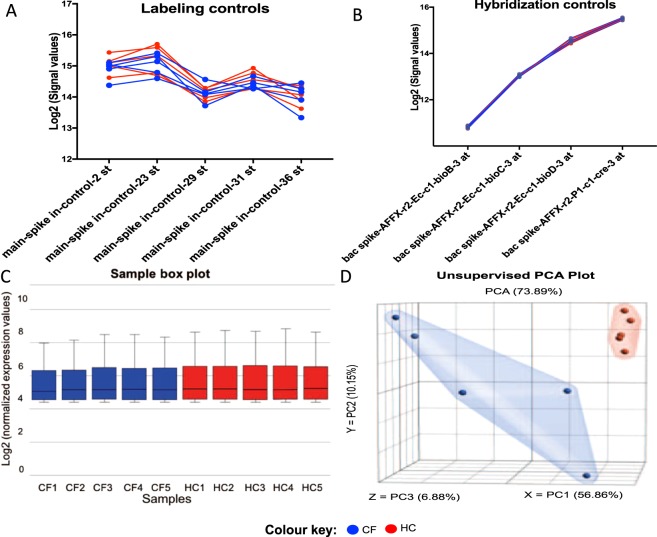
Quality assessment of miRNA microarray profiling. Each sample is represented in blue (CF) or red (HC). (**A)** Signal values (log2) of synthetic miRNAs greater than 9.96 indicates the labeling procedure was successful and the lack of RNases in the samples. **(B)** The increasing signal values of hybridization probe sets corresponding to their increasing relative concentration is an indication that hybridization, wash, stain, and scan procedures were successful. **(C)** The Sample Box plot shows a similar distribution of normalized expression values in all samples indicating no obvious outlier sample. **(D)** Unsupervised principal component analysis graph of normalized miRNA array data. Each dot represents a study sample assigned to one of the experimental groups, which are highlighted in either blue or red. A clear segregation of CF and HC samples indicates differing expression of miRNAs between the two groups. The first 3 principal components (X, Y, and Z) explain 74% of all variation in the expression dataset.

The signal value of the control probe sets spiked in during array preparation were analyzed in all samples to evaluate the success of labeling and hybridization procedures. As shown in Fig. 1, all spike-in control labeling probe sets showed log2 signal values ≥9.96 (Fig. 1A) and the hybridization control probe sets for each CF and HC sample showed increasing log2 signal values corresponding to their increasing concentrations (Fig. 1B). These preliminary assessments confirmed the success of microarray processing for each sample (Thermofisher Scientific, USA). Further evaluation of the RNA-normalized miRNA expression datasets showed even distribution among biological replicates, which indicated no obvious outlier samples (Fig. 1C). Exploration of unsupervised principal component analysis using normalized miRNAs expression signatures showed that the CF and HC samples clustered distinctly (Fig. 1D), suggesting an underlying association between CF status and ECmiRNA expression signatures.

### Identification of CF-relevant ECmiRNAs

**Figure 2:**
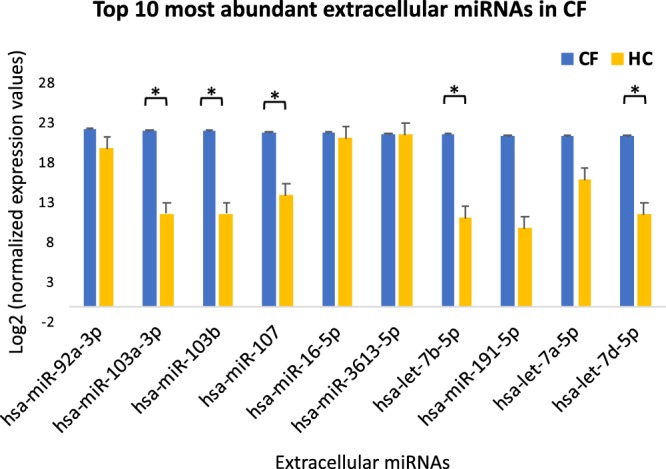
Several plasma ECmiRNAs are abundantly expressed in CF. The top 10 most abundant plasma ECmiRNAs in CF are represented on the x-axis and their normalized expression values are represented on the y-axis. Among the top 10 abundant ECmiRNAs, 5 showed significant differential expression between CF and HC samples (FDR < 0.05, |log2 FC|≥ 2). Significance was estimated using t-tests. * indicates *P* < 0.05.

**Figure 3:**
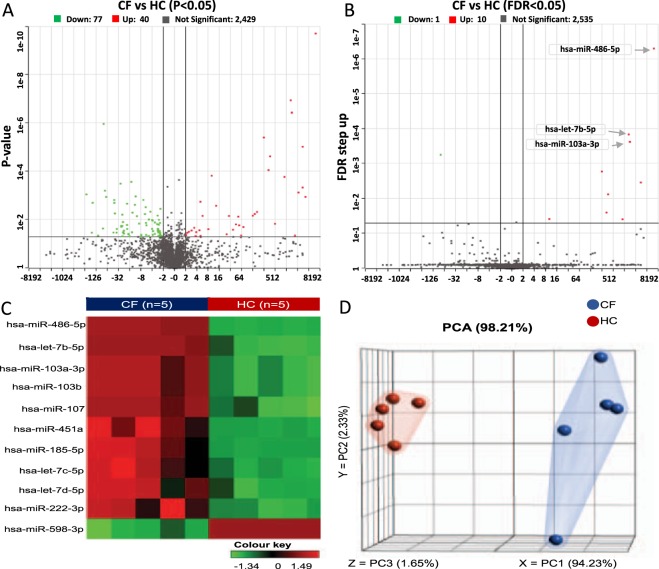
Plasma ECmiRNA expression signatures differ in CF and HC. (**A**,**B)** Volcano plot showing miRNAs differentially expressed in CF compared HC samples using p < 0.05 and FDR < 0.05, respectively. The expression value of each miRNA is represented on the vertical axis and the fold change differences are shown on the horizontal axis. **(C)** Hierarchical clustering showing differential expression of miRNAs in CF and HC samples. Red indicates high relative expression, and green indicates low relative expression. **(D)** Principal component analysis graph of the 11 differentially expressed miRNAs. Each dot represents a study sample assigned to one of the experimental groups, which are highlighted in either blue (CF) or red (HC). The 3 principal components (X, Y, and Z) plotted for the 10 samples explain 98% of the expression variance due to CF.

In order to identify the ECmiRNAs differentially expressed between CF and HC samples, the high-quality microarray datasets were mapped to miRbase mature miRNA annotation (v20) and quantified with Partek Quantify to Annotation model. The expression signatures of 2,546 miRNAs in the miRbase registry were captured. Among these, the top 10 most abundant ECmiRNAs in CF were identified (Fig. 2). miR-92a-3p exhibited the highest expression level in CF, but the levels were not significantly different from those in the HC samples. Intriguingly, 5 of the top 10 abundantly expressed ECmiRNAs were also significantly differentially expressed (FDR < 0.05, log2 FC≥2) between CF and HC samples (Fig. 2). By employing a liberal significance threshold (F-test, p < 0.05, log2 FC≥2), 117 (4.6%) miRNAs were identified as differentially expressed between CF and HC samples (Fig. 3A, Table S1). Of these, 40 (34.2%) were overexpressed in CF while 77 (65.8%) were underexpressed in CF compared to HC. To increase the discriminatory accuracy of identifying differentially expressed miRNAs influenced by CF, we corrected for multiple testing using a more stringent significance threshold (FDR < 0.05, log2 FC≥2). At this threshold, 11 miRNAs were found to be differentially expressed between CF and HC samples (Fig. 3B). Among these, 10 miRNAs were overexpressed in CF and one miRNA was underexpressed. The overexpressed miRNAs included hsa-miR-486-5p, 3 family members of let-7 (hsa-let-7b-5p, hsa-let-7c-5p and hsa-let-7d-5p), hsa-miR-103a-3p, and other miRNAs, while hsa-miR-598-3p was underexpressed in CF. Further hierarchical clustering and PCA plotted using the 11 most variable miRNAs showed clear segregation of the CF and HC samples (Fig. 3C,D, respectively). These 11 miRNAs were prioritized for functional analyses.

### RT-qPCR validation of ECmiRNAs differentially expressed in CF

**Figure 4:**
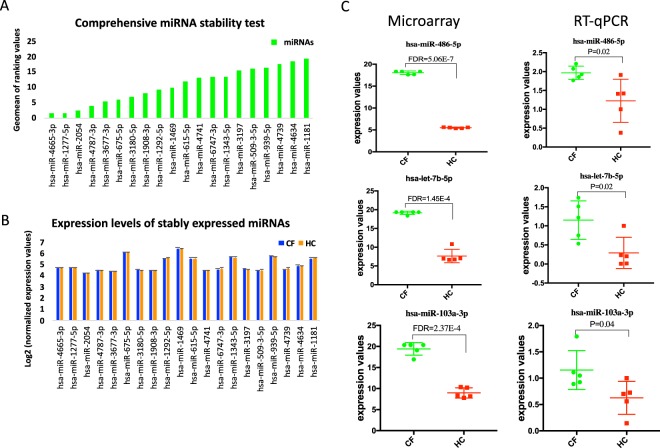
Differentially expressed miRNAs validated by RT-qPCR. (**A**) The results of miRNA stability testing are illustrated. Smaller ranking values correspond to miRNAs with high stability. hsa-miR-4665-3p was identified as the most stable candidate to use as an endogenous control for RT-qPCR. **(B)** Bar chart showing corresponding normalized microarray expression value for each miRNA tested. **(C)** Expression profiles of 3 validated miRNAs (hsa-miR-486-5p, hsa-let-7b-5b and hsa-miR-103a-3p) captured via microarray and RT-qPCR are shown. The RT-qPCR results confirmed that the differentially expressed miRNAs detected by microarray exhibit significantly higher expression levels in CF compared to HC subjects. Significance was estimated using t-tests. * indicates P < 0.05.

Using the results of our high-throughput microarray miRNA profiling, we first performed stability testing using the expression signatures of 20 least variable ECmiRNAs between CF and HC to identify the most stable ECmiRNA (Fig. 4A,B). The results showed hsa-miR-4665-3p had the smallest ranking value, which corresponds to the most stable candidate to use as an endogenous control for RT-qPCR (Fig. 4A). We selected the top three differentially expressed ECmiRNAs (hsa-miR-486-5p, hsa-let-7b, and hsa-miR-103a-3p) between CF and HC samples for validation via RT-qPCR. The analysis was performed using an independent cohort of CF and HC samples (Table 1). All three ECmiRNAs were significantly (p < 0.05) overexpressed in CF (Fig. 4C), confirming the results from the microarray screening.

### Identification of miRNAs hubs and functional enrichment analysis of their targets

**Figure 5:**
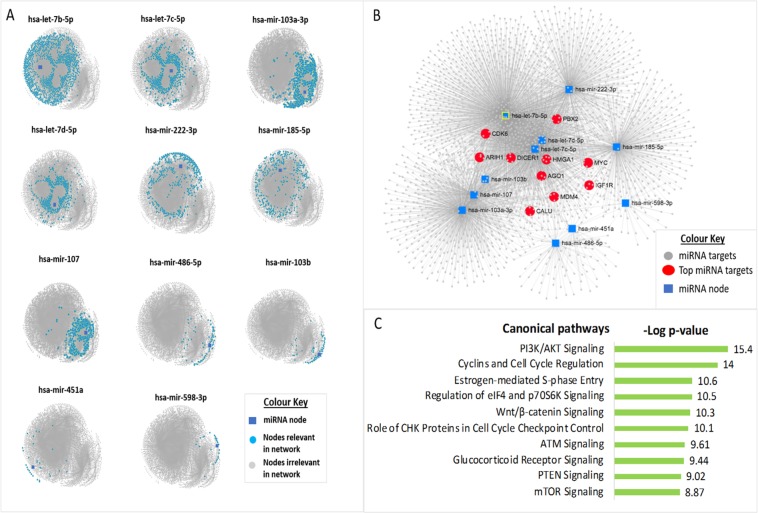
Identification of miRNA hubs and pathway enrichment of their targets. The differentially expressed miRNAs and their experimentally validated mRNA targets are illustrated in network interaction graphs. Each node represents a miRNA or mRNA. **(A)** Hubs of the 11 differentially expressed miRNAs are shown in individual networks. Nodes interacting with the miRNA are highlighted in blue. As shown, hsa-let-7b had the highest interaction (blue dots), indicating it is the most important in the network, whereas has-miR-451a had the lowest node interaction. **(B)** The top 10 genes (red node) and 11 dysregulated ECmiRNAs in CF (blue node) are highlighted in the network interaction graph. *HMGA1* is the most important gene in the network based on ranking highest in node degree and betweenness, whereas *PBX2* ranked lowest among the top 10 targets depicted (see Table 2). **(C)** The top 10 canonical pathways (non-disease) significantly influenced by the validated miRNA targets are represented in a bar chart (adjusted p < 0.05). Among these, PI3K/AKT was the most enriched pathway with an enrichment score of 15.4.

We employed the miRNet algorithm to identify experimentally validated mRNA targets of the 11 differentially expressed miRNAs in CF and to characterize their functional relevance. In total, 2,505 unique targets for the miRNAs were retrieved (Table S2). As shown in Fig. 5A, analysis of the network interaction graph showed that hsa-let-7b-5p was the most important hub in the network as it interacted with more nodes, with the highest node degree and betweenness compared to other nodes, while hsa-miR-451a had the lowest node degree (Table 2). Among the 2,505 targets, we depicted the top 10 miRNA-gene targets based on the results of their node degree and betweenness (Fig. 5B). As shown in Table 2, the top target list was dominated by genes involved in miRNA biogenesis and gene regulation. *MYC*, which encodes a protein crucial for gene expression, cell cycle progression, cell proliferation, and apoptosis, was one of the most important target hubs in the network. *DICER1*, which is known to play a crucial role in the biogenesis of miRNAs, was also one of the top 10 targets (Fig. 5B). We further performed canonical pathway analysis (non-disease) to elucidate the biological relevance of the miRNA targets. We identified several significant pathways (adjusted p < 0.05) influenced by the miRNA targets (Table S3). The top significant canonical pathways were primarily associated with signal transduction, including pathways such as PI3K/Akt signaling, Wnt/β-catenin signaling, glucocorticoid receptor signaling, and mTor signaling (Fig. 5C).

**Table 2:** Top miRNAs and genes in the network interaction.

Node	Degree	Betweenness
hsa-let-7b-5p	1215	1912155
hsa-let-7c-5p	516	437494.1
hsa-mir-103a-3p	453	667385
hsa-let-7d-5p	394	226216.3
hsa-mir-222-3p	394	716252.1
hsa-mir-185-5p	359	660043.5
hsa-mir-107	300	295251.4
hsa-mir-486-5p	67	122272.2
hsa-mir-103b	61	104926.3
hsa-mir-451a	31	48759.1
hsa-mir-598-3p	25	45443.85
*HMGA1*	6	34668.45
*MYC*	6	31210.28
*DICER1*	6	26774.36
*ARIH1*	6	25835.22
*AGO1*	6	25835.22
*CALU*	5	12963.25
*CDK6*	5	30866.04
*IGF1R*	5	20690.76
*MDM4*	5	12963.25
*PBX2*	5	18718.67

## Discussion

ECmiRNA play a crucial role in cell-to-cell communication and have shown great promise as non-invasive biomarkers in a wide range of diseases^20,26^, but knowledge of their abundance and functional relevance in CF remains poorly understood. In this study, we employed high-throughput microarray technology to identify differentially expressed extracellular miRNAs in CF compared to HC, and to elucidate the functional relevance of their mRNA targets. To our knowledge, no previous studies have reported plasma miRNA expression levels in patients with CF. Our results showed that several miRNAs are abundantly expressed in the extracellular milieu of CF patients, and that the top differentially expressed miRNAs targeted genes are involved in crucial biological processes, as well as miRNA biogenesis.

We identified 11 ECmiRNAs whose expression levels differed significantly between CF patients and HC. Among these, 10 miRNAs were overexpressed while only hsa-miR-598-3p was underexpressed in CF (Fig. 3B). In CF, altered expression of several miRNAs has been reported in epithelia^7–9^ and blood cells^12^. Although there is sparse literature on dysregulated ECmiRNAs in CF, a study that profiled circulating serum ECmiRNA levels in CF patients via qPCR array of 84 miRNAs identified a combination of miRNAs (miR-122, miR-25, miR-21) with diagnostic potential for CF liver disease^25^. In contrast to that study, we did not find significant differential expression of these three miRNAs between CF and HC plasma samples. This discrepancy could be explained by the fact that ECmiRNAs levels are influenced by sample source/type and different pathophysiological conditions, including disease progression^20,26^. Indeed, using hierarchical clustering and principal component analysis graphing, we found the expression signatures of 11 different ECmiRNAs that clearly segregated CF patients from the HC (Fig. 3C,D, respectively).

miR-486-5p was the most significant differentially expressed ECmiRNA between the CF and HC plasma samples (Fig. 3B). miR-486-5p is known to play a crucial role in hematopoietic cell differentiation via regulation of FOXO and AKT expression^27^, and its aberrant expression in plasma has been reported in numerous cancers^28,29^. Increased plasma expression levels of miR-486-5p was observed and demonstrated as a biomarker in both gastric^28^ and pancreatic cancers^29^. Similarly, we found striking elevated levels of miR-486-5p in CF plasma compared to HC by microarray. The results were subsequently validated with RT-qPCR using a unique cohort of CF patients and HC (Fig. 4C). Further study is encouraged to investigate the potential role of miR-486-5p in CF.

Additionally, we found three members of the let-7 (lethal-7) miRNA family (hsa-let-7b-5p; hsa-let-7c-5p; hsa-let-7d-5p) were significantly differentially expressed between CF and HC plasma samples. Let-7 was one of the earliest discovered miRNAs in humans. The Let-7 family comprises of several miRNAs that share the same highly conserved seed sequence, suggesting their targets and function may be similar across a diverse range of animal species^30^. For example, we found the three differentially expressed let-7 miRNAs share several similar targets (Table S2), including *AGO1*, *DICER1*, and *HMGA1*, which are crucial for many biological processes, particularly miRNA biogenesis and gene expression^30^. These three mRNA targets were also among the top 10 targets, with the highest node degree and betweenness in the interaction network (Table 2). Interestingly, we also identified three members of the let-7 family miRNAs (hsa-let-7a-5p; hsa-let-7b-5p and hsa-let-7d-5p) to be among the top 10 most abundant ECmiRNAs in CF (Fig. 2). Although, hsa-let-7a-5p was of high abundance in CF, the expression levels were not significantly different compared to the HC samples (Fig. 2). Notably, hsa-let-7b-5p was equally of high abundance (Fig. 2) and had the highest interaction in the network analysis (Fig. 5A).

Let-7b is one of the most studied of the let-7 miRNA family and its functional role has been characterized in some cell types. For example, let-7b was demonstrated to regulate immunity-related genes such as* IL6* and *TNF* in monocytes and *SERPINE1* in lipopolysaccharide-induced macrophages^31^, implying that let-7b dysregulation in cells may impair immune responses to pathogenic agents. Let-7b was also demonstrated to be involved in regulating the activation of hepatic stellate cells by interacting with lin28 in human alcoholic liver disease^32^. In plasma, aberrant let-7b expression levels has been implicated in many diseases including breast cancer^33^, prostate cancer^34^, hepatitis C^35^, and ischemic stroke^36^. The significant elevated expression levels of let-7b-5p found in CF plasma in this study, as well as its identification as the most important node in our network analysis, underscores the need to investigate its functional role in the extracellular spaces of CF patients.

Additionally, we found three members of the 103/107 miRNA family (hsa-mir-103a-3p; hsa-mir-103b; hsa-mir-107) were significantly overexpressed in CF plasma compared to the HC samples. Previous studies have shown that miR-103/107 are involved in several biological processes including angiogenesis^37^, apoptosis^38^, autophagy^39^, glucose homeostasis, and insulin sensitivity^40^. Members of the 103/107 miRNA recognize similar targets by virtue of having the same seed sequence (GCAGCAU). Their aberrant expression has been implicated in human diseases such as Alzheimer^41^, breast cancer^42^, diabetes^43^, obesity^44^, and schizophrenia^45^. These miRNAs regulate the expression of genes involved in crucial biological pathways. For example, it has recently been demonstrated in preadipocytes that by targeting Wnt3a, miR-103/107 aggravates endoplasmic reticulum stress mediated apoptosis and inhibits the canonical Wnt/β-catenin signaling pathway^38^. Although in CF cells, the role of miR-103/107 is unknown, dysregulated Wnt/β-catenin signaling has been reported in CF epithelial cells^5,46^. With the results of our functional analysis also identifying the Wnt/β-catenin signaling pathway as one of top significantly enriched canonical pathways in CF (Fig. 5C), further studies to investigate the regulatory role of the miR-103/107 in CF cells are warranted.

By modulating the expression of their target genes, miRNAs can contribute to biological pathway dysfunction, a common feature of many human diseases, including CF^5^. As shown in Fig. 5C, canonical pathway analysis of the genes targeted by the 11 differentially expressed miRNAs in CF identified several significantly enriched pathways. Among these, the top 10 enriched pathways were primarily signal transduction pathways and included mTOR signaling, PI3K/Akt signaling, and Wnt/β-catenin signaling (Fig. 5C). This is consistent with our recent report indicating several signaling pathways are defective in CF^5^. Interestingly, inhibition of the PI3K/Akt/mTOR signaling pathway leads to increased expression and stability of CFTR, which suggests it is a potent therapeutic target for CF^47^. With recent research demonstrating that plasma-derived extracellular vesicles can be engineered to deliver miRNAs to recipient cells where they can alter the expression of their target genes and subsequently mediate biological processes^22^, it is conceivable miRNAs may not only serve as biomarkers but as therapeutic targets to modulate dysfunctional pathways in CF.

In summary, we utilized microarray technology to identify and characterize the functional relevance of aberrantly expressed extracellular miRNAs in CF patients. Until now, there was limited literature about ECmiRNA abundance and their altered expression in CF. For the first time, using plasma samples, we showed that several ECmiRNAs are differentially expressed between CF and HC samples. We showed that the top validated targets of the dysregulated miRNAs are genes involved in miRNA biogenesis and gene expression. In addition, canonical pathway analysis indicated that the dysregulated miRNAs target genes were enriched mostly in signal transduction pathways. Overall, our findings support previous studies by demonstrating that plasma ECmiRNA expression profiles are influenced by disease states. These results indicate that ECmiRNAs may be clinically relevant in CF. In CF, the substantial clinical variation seen among patients warrants a need to identify novel molecular markers that can define the disease states and therapeutic responses. Future studies using larger sample sizes are encouraged to investigate the utility of ECmiRNAs as biomarkers for CF and its phenotypes.

## Methods

### Sample subjects and plasma processing

**Figure 6:**
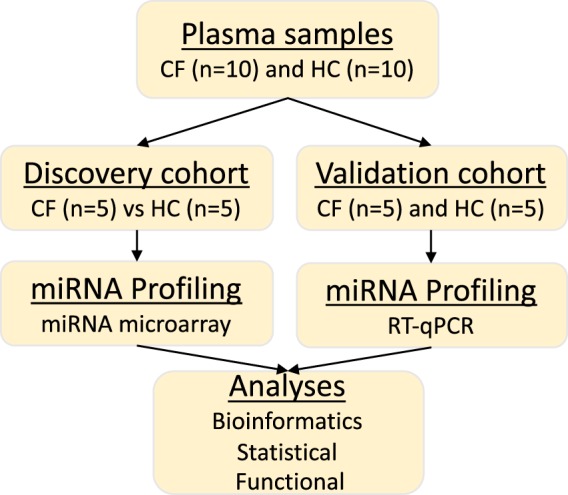
Illustration of the workflow. A total of 20 blood plasma samples collected from CF patients (n = 10) and HC (n = 10) were profiled to examine differential expression of circulating extracellular miRNAs. All CF patients (p.Phe508del homozygotes) were diagnosed based on *CFTR* genotype and/or sweat chloride test (≥60 mmol/L) and were negative for *Pseudomonas aeruginosa* infection at the time of recruitment. Half (n = 5) of the samples in each group (CF and HC) were analyzed on an Affymetrix GeneChip miRNA 4.0 array during discovery and the other half were used for validation with RT-qPCR. Differentially expressed miRNAs were identified using Partek Gene-Specific Analysis algorithm and the functional relevance of their mRNA targets explored with the miRNET tool.

Blood plasma isolated from a total of 10 CF and 10 healthy control (HC) subjects were utilized for this study. All the CF samples (homozygous for DF508del *CFTR* mutation) were collected from patients recruited at Ann & Robert H. Lurie Children’s Hospital of Chicago. The study was approved by the Institutional Review Board (IRB# 2015-400) and informed consent was obtained from the subjects and/or their parents or legal guardians. All methods were performed in compliance to the institutional guidelines and regulations. All CF subjects were diagnosed based on pilocarpine iontophoresis sweat test (sweat chloride ≥60 mmol/L) and/or *CFTR* genotype, as previously described^48,49^. Other clinically relevant variables such as pancreatic function status, mucoid *Pseudomonas aeruginosa* infection status, and the forced expiratory volume in 1 second (FEV_1_) % predicted were recorded for each CF patient at the time of sample collection. The 10 HC plasma samples were acquired from Cellular Technology Limited (CTL, USA) and tested negative for common pathogens in accordance with the manufacturer’s protocols (Fig. 6).

Half of the CF and HC samples (5 samples per group) were used for comparison during the discovery and validation phases. For each CF sample, peripheral blood was drawn into BD Vacutainer^®^ Mononuclear Cell Preparation Tube (Beckon Dickinson, USA) and centrifuged at 1,700 × g for 20 min at 20 °C to separate cells from plasma. The supernatant was then transferred to a new conical tube, centrifuged at 1300 g for 10 min at 4 °C to pellet residual cells, and ~0.5 mL of supernatant was aliquoted in into new vials and frozen at 20 °C until further processing.

### RNA isolation

A total of 100 μL plasma collected from each sample was mixed with 400 μL TRIzol Reagent (Invitrogen Life Technologies, USA), incubated for 5 min at room temperature, and utilized for RNA extraction using the RNA Clean & Concentrator kit according to manufacturer’s recommendation with brief modification (ZymoResearch, USA). Prior to the addition of chloroform and phase separation, each plasma sample used in the validation phase was spiked with 4.2 × 10^8^ copies of *C*. *elegans* synthetic miRNA (cel-miR-39) in accordance with the manufacturer’s recommendation (Qiagen, USA). In-column DNASE I treatment was performed for all plasma samples prior to RNA elution according to the manufacturer’s protocol (ZymoResearch, USA). Plasma total RNA was eluted in 10 μL nuclease-free H_2_O and stored at −80 °C.

### miRNA microarray profiling

An Affymetrix Genechip miRNA 4.0 array was used according to the manufacturer’s instruction (Thermofisher Scientific, USA) to identify plasma ECmiRNAs differentially expressed between CF (n = 5) and HC (n = 5) samples. For each sample, 8 µL of plasma RNA was labelled using the FlashTag Biotin RNA Labelling kit and spiked with synthetic control miRNAs for accessing labelling performance. Hybridization was performed by injecting the labelled samples, which were mixed with hybridization cocktail (130 μL total volume), into the 100 Format miRNA microarrays and subsequent incubation in a 48 °C hybridization oven with continuous agitation at 60 rpm for 16 hrs. The microarrays were then washed and stained with the Genechip Fluidics Station 450 using a locally installed miRNA 4.0 protocol. The washed and stained arrays were then scanned using the Affymetrix Genechip 3000 Scanner (Thermofisher Scientific, USA).

### RT-qPCR validation

The other half of the CF (n = 5) and HC (n = 5) samples, representing a unique cohort, was utilized for RT-qPCR to validate the expression levels of selected differentially expressed miRNAs via the qScript MicroRNA System (Quantabio, CA, USA). Briefly, the qScript microRNA cDNA Synthesis kit (Quantabio, CA, USA) was used in a polyadenylation step to prepare plasma RNA samples, spiked with *cel*-miR-39 (Qiagen, USA), for cDNA synthesis. Next, qScript Reverse Transcriptase and other reagents were added to convert the poly(A) tailed miRNAs into cDNA using an oligo-dT adapter primer with a unique 5′ end sequence according to the manufacturer’s recommendation. RT-qPCR was then performed using the PerfeCTa SYBR Green Kit (Quantabio, USA) with an automated Fast SYBR Green protocol in a 7500 RT-PCR System (Thermofisher Scientific, USA). The 15 µL PCR mixture included 7.5 μL PerfeCTa SYBR Green SuperMix (2×), 0.5 μL mature miRNA sequence (Integrated DNA Technologies, USA) as forward primer, 0.5 μL PerfeCTa universal reverse primer, and 2 μL cDNA. All reactions were performed in duplicate.

As there is currently no consensus RNA to use as endogenous control for the normalization of ECmiRNA expression data^50^, we selected 20 miRNAs in the microarray dataset with low coefficients of variance in all biological replicates and performed RNA stability testing with RefFinder based on four algorithms (geNorm, Normfinder, BestKeeper, and the comparative Delta CT) to identify the most likely candidate to use as an endogenous control for normalization^51^. The expression value of synthetic cel-miR-39, which was spiked in during RNA purification, was used as the exogenous control for normalization and the relative expression levels of miRNAs were calculated using the 2−ΔΔCt method.

### Statistical and bioinformatics analysis

Microarray data were assessed for quality using Transcriptome Analysis Console software (v.4.0.1). Arrays with log2 signal value of the spiked-in controls ≥9.96 indicated a successful labeling protocol and a lack of RNases in the RNA sample. For the hybridization procedure, success was achieved if the signal value of controls corresponded with their increasing concentration (Thermofisher Scientific, USA). The high-quality probe cell intensity (CEL) files were then imported into Partek Flow installed in a local storage area network (SAN). Robust Multi-Chip Analysis (RMA) was enabled for microarray data background correction, quantile normalization, and summarization prior to alignment to the human reference genome (hg19) with Bowtie (v1.0.0). The miRbase Mature MicroRNA (v20) was used as the reference index and annotation model. The Partek Quantify to Annotation model, with a minimum feature-read overlap of 100%, was used for estimating miRNA abundance. Differential miRNA expression analysis was performed with the Gene-Specific Analysis algorithm (Partek Inc, USA). Mature ECmiRNAs meeting a significance threshold of false-discovery rate (FDR) < 0.05 with at least 2-fold change (FC) difference were considered to be differentially expressed and prioritized for functional analysis.

Clinical variables were analyzed using IBM SPSS Statistics for Windows (version 24). Mean and standard deviation were used for normally distributed data. A t-test was used to compare the groups. *P* < 0.05 was considered significant.

### Identification of miRNA targets, hubs, and functional analyses

The miRNET tool, which incorporates data from 11 databases (TarBase, miRTarBase, miRecords, miRanda, miR2Disease, HMDD, PhenomiR, SM2miR, PharmacomiR, EpimiR, and starBase)^52^, was used to identify validated mRNA targets for the differentially expressed miRNAs and the important hubs in their network. Nodes with high degrees (number of connections with other nodes) and betweenness (shortest path going through the nodes) corresponded to important hubs in a network and were prioritized in network analyses. Functional analysis was performed with the IPA tool (Qiagen, CA, USA) using the validated miRNA targets to identify significantly enriched (adjusted p < 0.05) canonical pathways (non-disease specific).

### Ethics approval and consent to participate

The study was approved by the Institutional Review Board of the Ann & Robert H. Lurie Children’s Hospital of Chicago, USA (IRB# 2015-400) and written informed consent was obtained from subjects or parents/legal guardians.

## Supplementary information

Supplementary Tables

## Data Availability

The datasets generated during and/or analyzed during the current study are available in the Gene Expression Omnibus (GEO) repository, and can be accessed with accession number: GSE135119.
